# Supplementary material for: Comparative Analysis of the Upper Respiratory Bacterial Communities of Pigs with or without Respiratory Clinical Signs: From Weaning to Finishing Phase
Source: Biology (Basel). 2022 Jul 26;11(8):1111. doi: 10.3390/biology11081111 (PMC9330314; doi:10.3390/biology11081111)
Supplement: Supplementary file 1 [file biology-11-01111-s001.zip › biology-1805308-supplementary.pdf]

Supplementary Table S1. Prophylactic use of antibiotics used in feed during the nursery and finishing phases.

**Phase**	**Antibiotic**	**Treatment time (days of age)**
Nursery	Tiamulin + norfloxacin	21-38
	Doxycycline + colistin	39-54
	Tilmicosin	55-71
Finishing	Florfenicol	72-81
	No antibiotic treatment	82-104
	Tiamulin	105-114
	Tylosin	115-132
	Florfenicol	133-142
	Tylosin	143-160
	Tiamulin + doxycycline	161-170
	Tylosin	171-188

Supplementary Table S2. Number of sequences after all filtering steps in each sample.

Sample	Group	Sampling Type	Number of sequences
S01	Asymptomatic-T1	Nasal	91423

S02	Asymptomatic-T1	Nasal	91805
S03	Asymptomatic-T1	Nasal	114077
S04	Symptomatic-T1	Nasal	52710
S05	Symptomatic-T1	Nasal	79801
S06	Symptomatic-T1	Nasal	82515
S07	Asymptomatic-T1	Laryngeal	83155
S08	Asymptomatic-T1	Laryngeal	82011
S09	Asymptomatic-T1	Laryngeal	76011
S10	Symptomatic-T1	Laryngeal	63342
S11	Symptomatic-T1	Laryngeal	104801
S12	Symptomatic-T1	Laryngeal	42289
S13	Asymptomatic-T2	Nasal	93070
S14	Asymptomatic-T2	Nasal	80536
S15	Asymptomatic-T2	Nasal	70728
S16	Symptomatic-T2	Nasal	80959
S17	Symptomatic-T2	Nasal	67798
S18	Symptomatic-T2	Nasal	63173
S19	Asymptomatic-T2	Laryngeal	68256
S20	Asymptomatic-T2	Laryngeal	45572
S21	Asymptomatic-T2	Laryngeal	86294

S22	Symptomatic-T2	Laryngeal	88570
S24	Symptomatic-T2	Laryngeal	56473
S25	T3	Nasal	90152
S26	T3	Nasal	82374
S27	T3	Nasal	68628
S31	T3	Laryngeal	60046
S32	T3	Laryngeal	91812
S33	T3	Laryngeal	88247
S36	T3	Laryngeal	77269
